# Efficacy and safety of pregabalin in eye pain: A systematic review

**DOI:** 10.1097/MD.0000000000032875

**Published:** 2023-02-10

**Authors:** Xiaohua Shen, Xingying Chen, Yanyan He, Hui Xu, Jia Zhu

**Affiliations:** a Department of Pathology and Molecular Medicine Center, Jiaxing Hospital of Traditional Chinese Medicine, Jiaxing University, Jiaxing, Zhejiang, China; b Forensic and Pathology Laboratory, Jiaxing University Medical College, Jiaxing University, Jiaxing, Zhejiang, China.

**Keywords:** eye pain, pregabalin, systematic review

## Abstract

The pregabalin is approved for the management of persistent pain. The aim of this study is to assess the advantages and disadvantages of the use of pregabalin in eye pain management.

**Methods:**

The PubMed, Cochrane Library, Embase, and Web of Science databases were searched until January 2022 for randomized controlled trials. Randomized, double-blinded trials comparing pregabalin with placebo in eye pain management were included. The primary outcome was visual analog scale or numerical rating scale at acute (24 hours) and chronic (≥7 days after surgery) timepoints. The secondary outcomes were analgesic medication requirements and pregabalin-related complications (nausea, vomiting, dizziness, and headache). We also compared the effect of pregabalin on dry-eye syndrome.

**Main results:**

Six relevant articles were identified that studied the use of pregabalin as pain relief for photorefractive keratectomy (n = 2), laser epithelial keratomileusis (n = 1), laser-assisted in situ keratomileusis (n = 1), eyelid surgery (n = 1), and dacryocystorhinostomy (n = 1). Pregabalin was associated with a significant reduction in pain scores (95% confidence interval = −0.41 [−0.76–−0.06]) 24 hours after surgical procedures. The data were insufficient to draw conclusions regarding dry eye symptoms. Because of the high heterogeneity of outcomes regarding adverse effects, there is no conclusion regarding the safety of pregabalin in eye pain.

**Conclusions:**

Pregabalin reduced acute eye pain but had no significant effect on long-term analgesia after ophthalmological surgery in adults. It had no effect on dry-eye symptoms after ocular surgery. Further studies on the safety of pregabalin in eye pain management are required to draw solid conclusions.

## 1. Introduction

Eye pain is one of the most distressing symptoms and complaints among ophthalmic patients. Basic eye history and eye examination are critical for diagnosing the cause of eye pain.^[1]^ Eye pain disorders are categorized into 2 groups: nociceptive pain and neuropathic pain.^[2]^ The former is usually caused by tissue inflammation, acute trauma, or surgery.^[3]^ Neuropathic pain usually arises from nervous system disease.^[4]^ These 2 kinds of pain are often found to coexist. Patients diagnosed with ocular symptoms and eye pain will further need pain management.

Pregabalin is a structural analog of γ-aminobutyric acid and is structurally related to gabapentin.^[5,6]^ Pregabalin is a third-line agent for eye pain management.^[7]^ It is efficient in controlling mild to moderate pain and has lower dose requirements and fewer dose-related complications.^[8,9]^ Although many studies have evaluated the efficacy and safety of pregabalin in pain management, there has been no systematic review of eye pain management using pregabalin. This review was conducted based on existing clinical randomized controlled trials (RCTs); the primary aim was to compare the efficacy and safety of pregabalin with those of placebo in acute and chronic eye pain control, and the secondary aim was to evaluate the effect of pregabalin on the relief of dry eye symptoms after eye treatment in adults.

## 2. Method

This study was conducted according to the recommendations of the Cochrane group and the PRISMA guidelines for systematic reviews and meta-analyses.^[10]^ Two authors (J.Z. and X.H.S.) performed the review and data extraction independently, and any discrepancy was resolved by consensus.

### 2.1. Search strategy

We searched for relevant studies in English, and there was no limitation on publication year. A systematic review of the relevant articles was conducted in 4 bibliographic databases, namely, PubMed, Cochrane Library, Embase, and Web of Science, until January 2022. Additionally, to ensure that most of the relevant studies were identified, key journals relevant to the topic were searched separately. We also searched Open Access Theses and Dissertations for any eligible unpublished studies. Pregabalin and eye pain were the search terms.

### 2.2. Study inclusion criteria and relevance screening

The duplicate records were removed, and a pair of authors (X.S. and X.C.) reviewed the titles and abstract of the articles independently. The exclusion criteria were as follows: the study focused on a pain model; animal experiments were used to study the effect of pregabalin; the study was a case report; the study was designed as an observational study; and all reviews and editorial comments. Moreover, the selected studies were included for analysis according to the following criteria: the study was designed as an RCT; the study focused on eye pain with no restriction to any specific age group; pain was measured as a primary outcome; the study compared pregabalin with placebo or another active treatment for pain; and the study was published in English.

For each reference identified by electronic search, 2 authors (X.S. and Y.H.) assessed eligibility according to the selection criteria defined above. References that met the inclusion criteria were retrieved in full-text format and further assessed independently by the same 2 authors. Reviewers resolved any disagreements by discussion to achieve consensus.

### 2.3. Data extraction

The selected studies were conducted independently by 2 reviewers (J.Z. and X.S.) based on the Cochrane Handbook for systematic reviews of interventions.^[11]^ Data, including study characteristics (design, longitude), participants’ characteristics (age, sex, and number of patients), treatment details (dose and dosing regimen), and efficacy outcomes, were extracted from all eligible studies using a constructed data extraction form. The primary outcome was acute pain (24 hours) and chronic pain (≥7 days after surgery). The secondary outcomes were side effects and ocular symptoms and signs.

### 2.4. Risk bias

The Cochrane Collaboration’s tool was used to assess the risk of potential bias in the selected studies. The quality of the included RCTs was assessed independently by 2 researchers (H.X. and Y.H.).^[11]^ The risk of bias tool requires reviewers to judge seven domains: methods of randomization; allocation concealment, blinding of the outcome assessors, blinding of the study participants, presence of incomplete data, selective reporting, and other sources. Each study was classified as having a high, unclear or low risk of bias. Only data from studies featuring a low or unclear risk of bias were included in our analysis.

### 2.5. Statistical analysis

Data analysis was completed independently by 2 authors (X.C. and Y.H.) using Review Manager v. 5.3 (The Nordic Cochrane Centre, Cochrane Collaboration, 2014, Copenhagen, Denmark). The mean difference and 95% confidence interval were used for continuous variables. The random-effects method and the fixed-effect method were used for studies with significant heterogeneity and for those without heterogeneity, respectively.

## 3. Results

### 3.1. Study selection and characteristics

**Figure 1.**
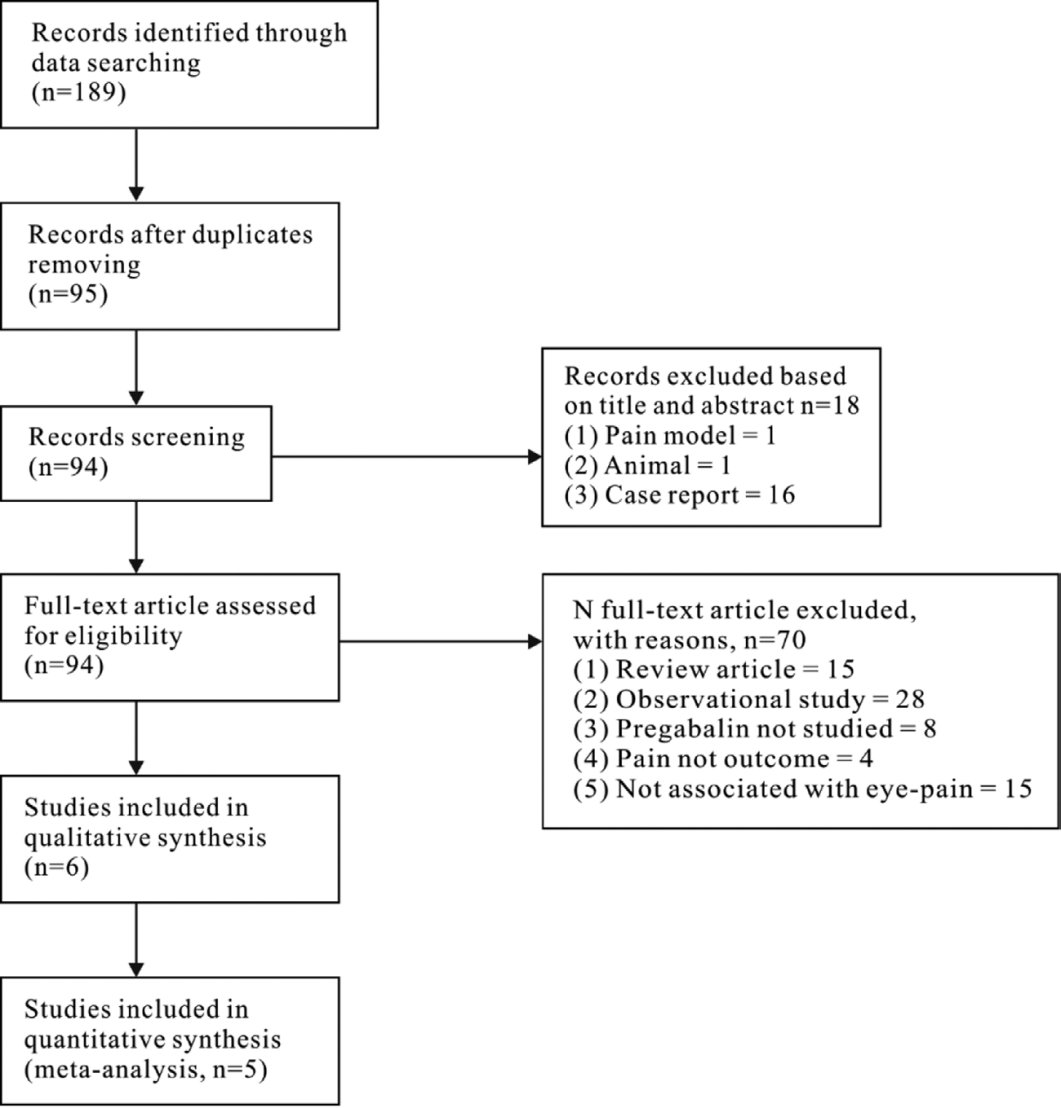
Flow diagram of the recruitment of studies to a systematic review of the safety and efficacy of pregabalin after eye surgery.

The flow chart of study identification is presented in Figure 1. A total of 189 publications relevant to search words were identified; 76 studies were deemed potentially eligible, and their full text versions were retrieved for full eligibility assessment. A total of 6 publications were included in the qualitative thesis (Table 1), with 244 patients in the pregabalin group and 238 participants in the placebo group. The study by Pakravan et al also used gabapentin as an intervention and had 50 patients in this group.^[12]^ The characteristics of each study are summarized in Table 1. They all in eye pain relief,^[13,14]^ and investigated the efficacy of pregabalin for laser epithelial keratomileusis (LASEK) and laser-assisted in situ keratomileusis surgery,^[12,15]^ investigated the efficacy of pregabalin for photorefractive keratectomy (PRK),^[16]^ investigated the efficacy of pregabalin for eyelid surgery, and^[17]^ investigated the efficacy of pregabalin for dacryocystorhinostomy. In general, the characteristics of the interventions in all 6 studies are described in Table 2. The studies by Paik et al and Galor et al did not present primary outcomes (pain scores at 24 hours and adverse effects), but they described the effect of pregabalin on chronic eye pain at 7 days, 3 months, and 6 months.^[13,14]^ The trial by Wei et al presented pain scores as the mean and range instead of the mean and standard deviation, which could not be used in the meta-analysis.^[16]^ Hence, we excluded these 3 papers from the meta-analysis but kept them in the systematic review.

**Table 1:** Study and patient characteristics for randomized controlled trials identified in systematic review regarding the efficacy and safety of pregabalin in eye pain.

Reference	Study funding	Country	Type of surgery	Sex F/M	Ages (yr)	Intervention/Comparator	Pain outcome	Dry-eye symptom	Adverse effect
Paik et al	National research foundation	Korean	Laser epithelial keratomileusis	40/40	Range 18–45	Pregabalin/placebo 40/ 40	Chronic pain	Yes	No
Galor et al	Department of Veterans Affairs etc	US	Laser-assisted in situ keratomileusis	22/21	Mean 37.8	Pregabalin/placebo 21/ 22	Chronic pain	Yes	Yes
Meek et al	None declared	US	Photorefractive keratectomy	70/60	Mean 34.86	Pregabalin/placebo 67/ 63	Acute pain	No	Yes
Wei et al	Synthes/AO foundation KLS Martin and AO foundation	US	Eyelid surgery	23/26	Mean 67.4	Pregabalin/placebo 26/ 23	Acute pain	No	Yes
Alimian et al	None declared	Iran	Dacryocystorhinostomy	31/49	Mean 43.25	Pregabalin/placebo 40/ 40	Acute pain	No	Yes
Pakravan et al	None declared	Iran	Photorefractive keratectomy	86/64	Mean 26.57	Pregabalin and gabapentin/placebo 50 and 50/ 50	Acute pain	No	No

**Table 2:** Invention characteristics for randomized controlled trials identified in systematic review regarding the safety and efficacy pregabalin after eye surgery.

Reference	Intervention	Route	Daily dose	Duration of administration	Comparator
Paik et al	Pregabalin (40)	Oral	150 mg twice per d	12 h before surgery, fellow by treatment for 15 d	Placebo (40)
Galor et al	Pregabalin (21)	Oral	150 mg twice per d	First dose prior to surgery, continue for 14 d	Placebo (22)
Meek et al	Pregabalin (67)	Oral	75 mg twice per day	2 h before surgery, continue every 12 h for 5 days	Placebo (63)
Wei et al	Pregabalin (26)	Oral	150 mg	15 min to 1 h prior to the eyelid surgery	Placebo (23)
Alimian et al	Pregabalin (40)	Oral	300 mg	1 h before the surgery	Placebo (40)
Pakravan et al	Pregabalin (50)	Oral	75 mg thrice per d	2 h after surgery and for 3 d	Placebo (50)
	Gabapentin (50)	Oral	300 mg thrice per d	2 h after surgery and for 3 d	

### 3.2. Methodological assessment and risk of bias assessment

**Figure 2.**
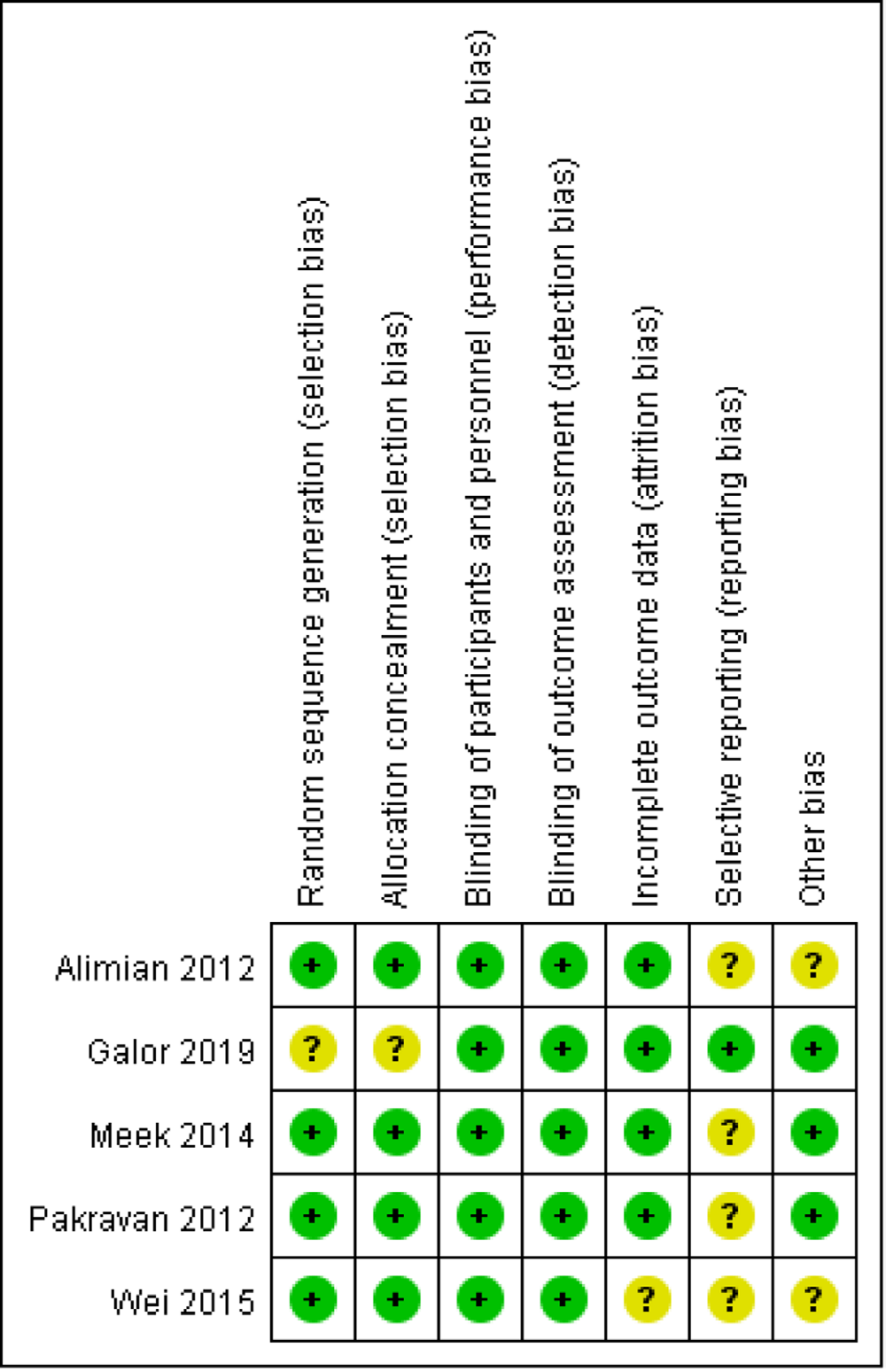
Quality summary of the pregabalin randomized controlled trials for each methodological quality item of each included study.

We used seven criteria outlined in the Cochrane Handbook for Systematic Reviews of Interventions to assess the quality of the RCTs included in our meta-analysis (Fig. 2). All the trials were placebo-controlled studies. Paik et al conducted a variable blocked randomization list for treatment assignments to ensure that treatment assignments were balanced, which was considered to be an unclear risk. Because of the lack of a study protocol for review, 4 studies were considered to have an unclear risk of report bias. One study was judged to be at an unclear risk regarding incomplete outcome data due to a lack of explanation about why 2 patients were not followed up. Two studies did not present baseline pain intensity, and they tended to have an unclear risk. All the studies were considered to have a low risk of methodological shortcomings. Five studies were identified as relevant, included in the meta-analysis and analyzed.^[12–17]^ Publication bias, statistically assessed using Begg and Egger tests, showed no significant bias (*P* = .42 and *P* = .36, respectively). Statistical heterogeneity for pain scores at 24 hours was moderate (*I*^2^ = 58%).

### 3.3. Pain-associated outcomes

**Figure 3.**
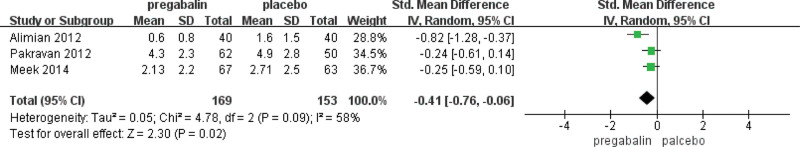
Pain scores 24 hours after surgery in pregabalin trials.

We summarized 6 trials on the use of different treatments to manage eye pain (Table 3). They studied acute and chronic eye pain by using the visual analog scale or numerical rating scale. Two trials focused on the chronic pain effect.^[17,18]^ A study by Wei et al showed that pregabalin reduced acute pain scores in histograms but had no specific data. Therefore, 3 trials were included in the quantitative analysis of ocular pain relief at 24 hours. Meta-analysis showed that pregabalin significantly reduced pain scores by 0.41 points (95% confidence interval −0.76–−0.06, *P* = .02, Fig. 3).

**Table 3:** Efficacy of pregabalin versus comparators in systematic review regarding the safety and efficacy pregabalin after eye surgery.

Reference	Primary outcome	Intervention	Comparator	Significant (*P* value)
Paik et al	Pain scores using VAS from baseline to post-operation 7 d, mean (SD)	0.15 (0.37)	1.5 (2.89)	*P* = .044
Galor et al	Ocular pain intensity using NRS at 3-mo, mean (SD)	0.85 (0.27)	0.27 (0.55)	Not significant
	Sf-MPQ sensory at 3-mo, mean (SD)	0.65 (1.23)	0.36 (0.66)	Not significant
	Sf-MPQ affective at 3-mo, mean (SD)	0.30 (0.66)	0.18 (0.39)	Not significant
	NPSI-eye at 3-mo, mean (SD)	2.7 (4.55)	1.86 (4.07)	Not significant
	Ocular pain intensity using NRS at 6-mo, mean (SD)	1.10 (1.48)	0.38 (0.97)	Not significant
	NPSI-eye, mean (SD)	2.81 (4.07)	3.14 (5.85)	Not significant
Meek et al	Pain intensity from VAS at day 1 AM, mean (SD)	6.32 (9.14)	10.22 (15.27)	Not significant
	Pain intensity from VAS at day 1 PM, mean (SD)	21.32 (21.95)	2.09 (25.03)	Not significant
	Pain intensity from VAS at day 2 AM, mean (SD)	23.49 (21.31)	29.27 (25.1)	Not significant
	Pain intensity from VAS at day 2 PM, mean (SD)	26.14 (22.79)	25.78 (26.66)	Not significant
	Consumption of total rescue pain medication at day 1, mean	1.7	2.4	<0.03
	Consumption of total rescue pain medication at day 2, mean	1.7	2.6	<0.025
Wei et al	Pain scores from VAS after 1–2 h of surgery, mean (range)	12.9 (0–73)	29.4 (0–94)	Patients in the pregabalin group reported pain score 5.5 points lower compared with the placebo group (*P* = .0307).
	Pain scores from VAS after 2–4 h of surgery, mean (range)	14.0 (0–48)	25.6 (0–76)	
	Pain scores from VAS after 8–12 h of surgery, mean (range)	10.8 (0–63)	20.6 (0–97)	
	Pain scores from VAS after 20–28 h of surgery, mean (range)	11.3 (0–80)	9.7 (0–69)	
	Pain scores from VAS after 36–48 h of surgery, mean (range)	11.0 (0–57)	2.1 (0–10)	
Alimian et al	Pain intensity from VAS at time of recovery, mean (SD)	3.2 (1.5)	5.1 (1.5)	<0.001
	Require for opioid to relieve pain, n (%)	7 (17.5%)	21 (52.5%)	*P* = .001
Pakravan et al	Pain intensity from VAS at day 1 AM, mean (SD)	Pregabalin: 4 (2.4) gabapentin 3.9 (2.4)	4.7 (3)	Not significant
	Pain intensity from VAS at day 1 PM, mean (SD)	Pregabalin: 4.3 (2.3) gabapentin: 4.3 (2.7)	4.9 (2.8)	Not significant
	Pain intensity from VAS at day 2 AM, mean (SD)	Pregabalin: 2.2 (1.8) gabapentin: 2.2 (2.3)	3.5 (3)	Not significant
	Pain intensity from VAS at day 2 PM, mean (SD)	Pregabalin: 2 (1.9) gabapentin: 2.1 (2.4)	2.7 (2.6)	Not significant
	Consumption of rescue pain medication, mean (SD)	Pregabalin: 7.9 (5.2) gabapentin: 9.0 (4.1)	10.3 (5.6)	Not significant

SD = standard deviation, Sf-MPQ = short form McGill pain questionnaire, NPSI = neuropathic pain symptom inventory-eye, NRS = numerical rating scale, VAS = visual analogue scale.

Paik et al and Galor et al also studied the chronic effect of pregabalin on pain relief.^[13,14]^ A study by Paik showed that patients took pregabalin 150 mg twice daily for 14 days. The ocular pain scores were significantly reduced compared with the placebo group for 1 week. While Galor’s study had no similar result, they showed that pregabalin did not have an effect on pain for up to 3 and 6 months. Two trials did not investigate pain at similar times, and 3 and 6 months was much longer than 1 week. Therefore, the chronic effect of pregabalin needs to be further studied to reach a solid conclusion.

Three trials investigated the effect of pregabalin in the consumption of rescue pain medication postoperatively. Meek et al showed that there was a significant decrease in the consumption of total rescue pain medication per patient on postoperative Days 1 and 2 in the pregabalin group.^[15]^ Pakravan et al reported a similar result, although the consumption of rescue medication in the pregabalin group did not reach statistical significance.^[12]^ Alimian et al reported that patients in the pregabalin group were less likely to require opioids than those in the placebo group.^[17]^

### 3.4. Ocular symptoms and sign-associated outcomes

Paik et al and Galor et al studied dry eye symptoms after keratomileusis.^[13,14]^ A trial by Galor et al showed that perioperative pregabalin did not reduce the frequency of dry-eye symptoms at a 6-month follow-up after laser-associated in situ keratomileusis. Paik evaluated dry-eye symptoms at 1, 3, and 6 months; there was no significant difference between the pregabalin and placebo groups. They both used the ocular surface disease index, dry eye questionnaire 5, and tear breakup time index to evaluate dry-eye symptoms (Table 4). Paik et al also tested corneal nerve regeneration by assessing the subbasal nerve plexus, and there was no significant difference between the 2 groups in nerve fiber density, fiber length and nerve branch density.

**Table 4:** Proportion of patients about ocular symptoms and signs.

Reference	Outcome measure	Intervention	Comparator	Significant
Paik et al	OSDI at 1-mo, mean	19.46	24.12	Not significant
	DEQ-5 at 1-mo, mean	8.27	8.54	Not significant
	Nerve fiber density, mean (SD)	8.40 (3.10)	7.70 (2.70)	Not significant
	Nerve fiber length, mean (SD)	3.18 (1.23)	2.60 (1.03)	Not significant
	Nerve branch density, mean (SD)	9.45 (4.25)	7.35 (2.42)	Not significant
Galor et al	DEQ-5 at 3-mo, mean (SD)	6.6 (3.9)	4.7 (4.4)	Not significant
	Change in DEQ5 from baseline, mean (SD)	1.1 (3.9)	0.4 (4.0)	Not significant
	OSDI at 3-mo, mean (SD)	11.9 (11.5)	11.0 (16.6)	Not significant
	Change in OSDI from baseline, mean (SD)	−0.01 (15.6)	2.0 (15.1)	Not significant
	TBUT at 6-mo, seconds, mean (SD)	8.36 (2.46)	9.05 (5.93)	Not significant
	Schirmer score at 6-mo, mm, mean (SD)	15.45 (8.17)	15.05 (8.64)	Not significant

DEQ-5 = dry eye questionnaire 5, OSDI = ocular surface disease index, SD = standard deviation, TBUT = tear breakup time.

### 3.5. Adverse effects

Adverse effect outcomes from pregabalin were reported by 4 of 5 retained studies (Table 5). Due to the heterogeneity of the study outcome of adverse effects, side effects were not included in the conclusion. However, the original results for each study were described in this review. Three trials reported that the number of adverse events in the pregabalin group was greater than that in the placebo group,^[14–16]^ and 1 trial had opposite results.^[17]^ However, these differences in each study were not statistically significant. In Galor’s study, they reported that subjects in the pregabalin group had a higher frequency of tiredness and dizziness than those in the control group (*P* < .05, Table 5). Alimian et al reported that patients in the pregabalin treatment group had a lower frequency of nausea (*P* < .05, Table 5). Nausea and dizziness were the most commonly reported individual adverse effects, often reported in between 3.8% and 12.5% and 7.7% and 29% of patients in the pregabalin group.

**Table 5:** Proportion of patients about adverse effects with pregabalin versus comparator in systematic review regarding the safety and efficacy pregabalin after eye surgery.

Reference	Primary outcome	Intervention	Comparator	Significant
Galor et al	Total side effect, n (%)	13 (62%)	10 (46%)	Not significant
	Tiredness, n (%)	8 (38%)	2 (9%)	*P* = .03
	Dizziness, n (%)	6 (29%)	1 (5%)	*P* = .05
	Headache, n (%)	3 (14%)	3 (14%)	Not significant
	Nausea, n (%)	1 (5%)	2 (9%)	Not significant
	Dry mouth, n (%)	3 (14%)	0 (0%)	Not significant
	Constipation, n (%)	3 (14%)	1 (5%)	Not significant
	Bloating, n (%)	3 (14%)	4 (18%)	Not significant
	High or elevated mood, n (%)	4 (19%)	1 (5%)	Not significant
Meek et al	Total adverse events, n	39	33	Not significant
	Dizziness/lightheadedness, n	10	2	Not significant
	Nausea, n	3	9	Not significant
	Somnolence, n	9	5	Not significant
	Rhinorrhea/ congestion, n	7	7	Not significant
Wei et al	Total adverse events, n (%)	9 (34.6%)	4 (17.4%)	Not significant
	Sleepiness, n (%)	4 (15.4%)	0	Not significant
	Dizziness, n (%)	2 (7.7%)	2 (8.7%)	Not significant
	Nausea, n (%)	1 (3.8%)	2 (8.7%)	Not significant
	Headache, n (%)	2 (7.7%)	0	Not significant
Alimian et al	Total adverse events, n (%)	6 (15%)	22 (55%)	Not described
	Nausea, n (%)	5 (12.5%)	17 (47%)	*P* = .03
	Vomiting, n (%)	1 (2.5%)	5 (12.5%)	Not significant

## 4. Discussion

Eye pain is a danger signal for ocular conditions, either in a “quiet eye or “red eye.”^[18,19]^ Many causes trigger eye pain, and neurologists must identify these causes. Moreover, these patients also need effective pain management to relieve pain. Increasing studies of the analgesic efficacy and safety of pregabalin in the management of pain have been investigated, and they included both acute and chronic pain.^[20–22]^ In our review, 6 relevant RCTs in eye pain were identified. Two clinical trials focused on postoperative pain (dacryocystorhinostomy and eyelid surgery). Four other trials studied corneal surgery, such as PRK, LASEK and laser-assisted in situ keratomileusis. They all investigated the effect of pregabalin on pain management, and 4 trials studied the adverse effects in the pregabalin and placebo groups. Therefore, we synthesized eye pain scores after treatment between the pregabalin and placebo groups from the included studies.

Pregabalin is an anticonvulsant medication approved for reducing neuropathic pain in various diseases.^[23–25]^ Pregabalin manages pain by diminishing hyperalgesia and central sensitization.^[26,27]^ Clinical trials have shown that pregabalin can relieve acute and persistent postoperative pain.^[28,29]^ Currently, oral administration of pregabalin has already been used in managing ocular pain,^[30]^ but there is no conclusion about the efficacy and safety of pregabalin in eye pain management. Our findings suggest that pregabalin may reduce acute eye pain within 24 hours after surgery (Fig. 3).

Orally administered pregabalin has maximal oral bioavailability and a half-life of 6.3 hours.^[31]^ Although effective pain control is dependent on the timing of analgesic administration, these trials orally administered pregabalin before or 1 to 2 hours after surgery. It seemed that the difference in dosing time did not affect the analgesic effect of pregabalin. Paik et al and Galor et al investigated the effect of pregabalin on chronic pain.^[13,14]^ Paik et al tested the pain intensity at 7 days postoperation, and pregabalin significantly reduced pain compared with placebo.^[13]^ However, Galor et al tested ocular pain at 3 and 6 months, and there was no difference in pain control, suggesting that a single administration of pregabalin may not be adequate to reduce long-term pain. Pregabalin was not able to suppress the entire afferent barrage of nociceptive signals or chemical mediators originating from the cornea and affect the central nervous system, leading to sensitization and chronicity of pain.^[32]^ However, they did not test at a similar time, and the 7-week and 3- or 6-month durations were quite different regarding the effect of analgesics. Hence, the effect of chronic pain management with pregabalin on cornel injury needs further study.

Except for the trials by Alimian et al and Wei et al, the other trials included ocular surgical procedures that are used to correct refractive error, including PRK, laser-assisted in situ keratomileusis surgery and LASEK, but only 2 trials reported ocular symptoms.^[13,14]^ These surgeries induce direct injury to the cornea and nerve endings of the treated corneal area.^[33]^ While visual outcomes after surgery tend to be excellent, a potential side effect of the procedures is the onset of dry eye symptoms.^[34,35]^ These studies used several measurements to test dry eye symptoms, such as ocular surface disease index, dry eye questionnaire 5, TUBT, and Schirmer scores. These 2 studies reported that there was no significant difference between the pregabalin and placebo groups at any point in the follow-up period. Paik et al also investigated the effect of pregabalin on corneal nerve regeneration, and they reported that there was no statistically significant difference between the pregabalin and control groups by assessing the subbasal nerve plexus, including nerve fiber length and nerve branch density. These 2 trials provided similar results regarding the relationship between pregabalin administration and the rate of dry eye symptoms at the 3- or 6-month follow-up after refractive surgery, which suggested that pregabalin may have no effect on dry eye symptoms after ocular surgery.

The data regarding the prevalence of adverse effects appeared relatively consistent, with dizziness nausea being the most common adverse effect of pregabalin.^[36]^ Due to the high heterogeneity of the included studies and the variability in the assessment of the adverse effect outcomes, pooling of the outcome data was not attempted, and the results are presented for each individual study (Table 5). Except for the study of Alimian et al, the other 3 studies reported that patients in the pregabalin group had a higher frequency of side effects, although the difference did not reach statistical significance.^[14–16]^ Patients had a higher rate of side effects prolonged with administration of pregabalin.^[37]^ A meta-analysis showed that administering pregabalin in doses over 300 mg before surgery significantly alleviated pain in the first 24-hour period after surgery, while increasing the dose significantly amplified the side effects resulting from pregabalin.^[38]^ However, Alimian and colleagues’ results showed that a single oral dose of pregabalin at 300 mg per dose is more effective than doses under 300 mg or in divided doses compared to other studies; these authors suggested that the type of surgery is an important factor and that in minor to moderate surgeries, pregabalin has its maximal effect without any side effects.^[17]^

There were several limitations to our study. First, the current review included only English language published studies, which induced bias if relevant studies were reported in other languages. Thus, more RCTs with larger sample sizes are urgently needed. Second, only 2 studies reported ocular symptoms, including dry eye and ocular fiber regeneration, after pregabalin administration. Third, the timing and dosage of pregabalin administration was inconsistent across trials, making it difficult to draw a conclusion about the safety of pregabalin in eye pain management. The optimal dose and best stage of pregabalin administration remain to be confirmed by future RCTs. In conclusion, this review found a paucity of data supporting the clinical use of pregabalin as a prophylactic or treatment option for eye pain.

## Author contributions

**Conceptualization:** Hui Xu.

**Data curation:** Xingying Chen.

**Formal analysis:** Yanyan He.

**Methodology:** Yanyan He.

**Project administration:** Xiaohua Shen.

**Supervision:** Jia Zhu.

**Writing – original draft:** Xiaohua Shen.

**Writing – review & editing: Jia Zhu.**
